# Skin Involvement and Pulmonary Hypertension Are Associated with Vitamin D Insufficiency in Scleroderma

**DOI:** 10.3390/ijms17122103

**Published:** 2016-12-14

**Authors:** Marco Atteritano, Domenico Santoro, Giorgio Corallo, Elisa Visalli, Michele Buemi, Antonino Catalano, Antonino Lasco, Alessandra Bitto, Francesco Squadrito

**Affiliations:** Department of Clinical and Experimental Medicine, University of Messina, 98125 Messina, Italy; santisi@hotmail.com (D.S.); gcorallo@hotmail.it (G.C.); elisigna@hotmail.it (E.V.); buemim@unime.it (M.B.); catalanoa@unime.it (A.C.); alasco@unime.it (A.L.); abitto@unime.it (A.B.); fsquadrito@unime.it (F.S.)

**Keywords:** immunomodulatory, pulmonary hypertension, systemic sclerosis, skin, vitamin D

## Abstract

Vitamin D status has been linked to immune system and autoimmune disorders; in fact, low levels of vitamin D are common in many autoimmune disorders. The aims of our study were to assess the prevalence of vitamin D insufficiency and the possible correlation with clinical parameters in systemic sclerosis (SSc). We recruited 40 patients (38 female and two male) with scleroderma and 40 healthy controls matched for age and gender. Demographic and clinical parameters were recorded and the 25-hydroxivitamin D3 serum levels were measured. Serum 25-hydroxivitamin D3 levels were significantly lower in patients with systemic sclerosis than in the control group. The prevalence of 25-hydroxivitamin D3 insufficiency was 50% in the patients and 22.5% in the control group. A statistically significant association was observed between the insufficiency of 25-hydroxivitamin D3 and skin involvement (*p* = 0.02) and echocardiography systolic pulmonary artery pressure >35 mmHg (*p* = 0.02). Our data show that the systemic sclerosis group has significantly lower serum 25-hydroxivitamin D3 concentrations compared to the control group; skin involvement and pulmonary hypertension are associated with vitamin D3 insufficiency.

## 1. Introduction

Systemic sclerosis (SSc) is a chronic autoimmune disorder of the connective tissue, characterized by vascular abnormalities and diffuse fibrosis of the several organs, such as kidney, esophagus, heart and lung. Increased synthesis and deposition of collagen is caused by a pathological activation of fibroblasts [1]. Transforming growth factor-β (TGF-β) is a central mediator of fibroblast activation in SSc [2], and regulatory T cells seemingly play an important role in skin fibrosis [3]. Vitamin D receptors (VDR) are expressed on natural killer cells, B and T lymphocytes, suggesting an effect on innate and adaptive immune responses [4,5]. Severe 25(OH)D_3_ deficiency as in genetically predisposed individuals impairs self-tolerance and immune responses by compromising the functions of dendritic cells, regulatory T cells, Th1 cells and B cells [6]. Recently Zerr et al. demonstrated that VDR is a negative regulator of fibroblast activation that interferes with the pro-fibrotic effects of TGF-β [7]. The 25(OH)D_3_ status has been linked to immune system and autoimmune disorders [8,9]; a high prevalence of low levels of 25(OH)D_3_ is common in many autoimmune disorders, such systemic lupus erythematosus (SLE) [10,11], rheumatoid arthritis (RA) [12,13,14,15], scleroderma [16], and also in non-rheumatic diseases, including autoimmune thyroid disease [17,18], multiple sclerosis [19], type 1 diabetes mellitus [20] and chronic renal failure [21]. We suppose that vitamin D may have a role in the complex clinical evolution of systemic sclerosis. The aim of our study was to estimate the prevalence of 25(OH)D_3_ insufficiency and correlate it with clinical parameters in SSc patients.

## 2. Results

**Figure 1:**
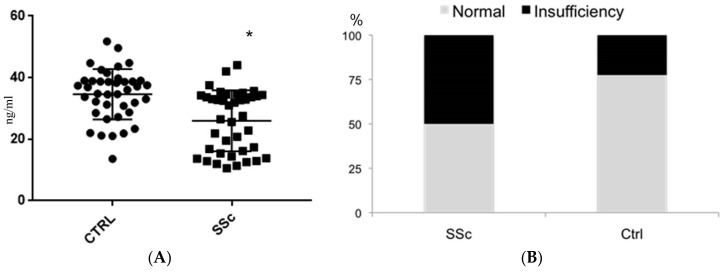
(**A**) The 25(OH)D_3_ serum levels in systemic sclerosis and control groups. * *p* = 0.0003; (**B**) Prevalence of 25(OH)D_3_ insufficiency in systemic sclerosis and control groups. *p* = 0.02.

**Figure 2:**
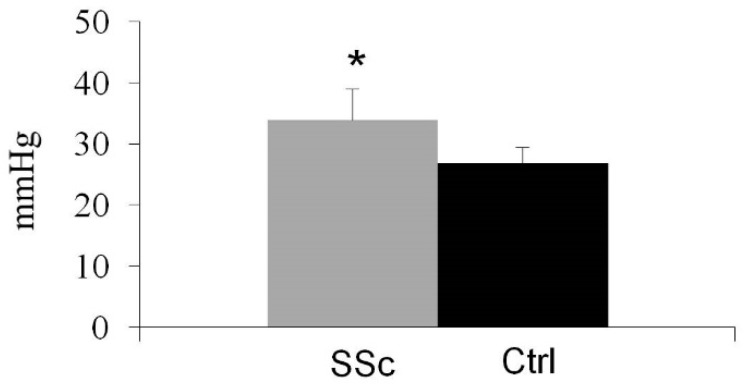
sPAP measurements in SSc and control groups. * *p* = 0.013.

In Table 1 reports the main demographic data of the participants. The two groups were comparable for gender (*p* = 1.00), age (*p* = 0.61) and Body Mass Index (BMI) (*p* = 0.26). The clinical features of patients with systemic sclerosis are shown in Table 2. The patients with systemic sclerosis have a 25(OH)D_3_ serum level statistically significantly lower than that of the control group (25.77 ± 12.84 vs. 35.08 ± 9.07; *p* = 0.0003) (Figure 1A). Insufficiency levels of 25(OH)D_3_ were observed in 20 of 40 patients with SSc (50%) and in nine of 40 healthy controls (22.5%), with a statistically significant difference (*p* = 0.02; Figure 1B). No subjects in either group had values of 25(OH)D_3_ <10 ng/mL. In SSc patients, the comparison between subjects with normal vitamin D serum levels and subjects with vitamin D insufficiency showed that the latter reported a higher Systolic Pulmonary Artery Pressure (sPAP) measurement (33.90 ± 10.60 vs. 26.80 ± 6.12 mmHg; *p* = 0.013) (Figure 2). Table 3 shows the associations between insufficiency of 25(OH)D_3_ and clinical parameters in scleroderma patients. A Rodnan skin score above 10 (*p* = 0.02) and systolic pulmonary artery pressure (sPAP) >35 mmHg (*p* = 0.02) from echocardiography were significantly associated with the insufficiency of 25(OH)D_3_. These associations were significant after adjusting for age, renal function and systemic hypertension. No other significant associations were found between 25(OH)D_3_ serum levels and the other clinical parameters assessed in SSc patients. In accordance with the Valentini index, the mean 25(OH)D_3_ in patients with a score ≥3 was 26.12 ± 11.13 ng/mL, and in patients with a score <3 it was 25.23 ± 12.15 ng/mL; the difference was not statistically significant (*p* = 0.81). The mean 25(OH)D_3_ in patients with a Medsger score >1 was 25.77 ± 12.84 ng/mL, and in patients with a Medsger score of 1 it was 24.42 ± 11.89 ng/mL; the difference was not statistically significant (*p* = 0.62). There were no differences in 25(OH)D_3_ serum levels between patients with and without hand calcinosis. In SSc patients, the 25(OH)D_3_ serum levels significantly correlated with Parathyroid Hormone (PTH) serum levels (*r* = −0.50; *p* < 0.05). The use of drugs, such as corticosteroids, antihypertensives, vasodilators, immunosuppressives or antiplatelet drugs, did not differ significantly between SSc patients with normal or insufficient levels of 25(OH)D_3_.

## 3. Discussion

There is a robust body of evidence showing the effect of vitamin D on the immune system [22,23,24,25]. Our results demonstrate that SSc patients have lower 25(OH)D_3_ serum levels compared to control subjects. Thus, our data are consistent with the current literature, which supports the evidence that patients affected by systemic sclerosis from different geographical locations, including Europe and Africa, have vitamin D insufficiency [26,27,28]. In the SSc group, we found a prevalence of insufficiency of vitamin D3 of 50%, significantly higher than in the controls, but lower than what has been previously reported in the northern Italian scleroderma population [28]. In our control group, we also found a lower prevalence of vitamin D insufficiency compared to previously published data, although this difference could be explained by the fact that the populations were not comparable [29].

Vitamin D3 or cholecalciferol is a steroid hormone that plays a role in calcium and bone metabolism. It is product in the skin by the photolysis of 7-dehydrocholesterol. Vitamin D3 undergoes two subsequent hydroxylations to form the active form of the vitamin. The first hydroxylation occurs in the liver to form 25-hydroxyvitamin D, 25(OH)D_3_, or calcidiol and subsequent transport to the kidney allows the second hydroxylation to form the active form 1-25-dihydroxyvitamin D_3_, 1,25(OH)_2_D_3_, or calcitriol. We supposed, after the exclusion of causes related to intestinal and/or renal disease and/or use of drugs, that skin thickening associated with capillary damage and rarefaction can result in reduced synthesis by UVB radiation in the epidermis [30]. To support this hypothesis, our results showed that vitamin D insufficiency is associated with skin involvement. Thirteen patients (32.5%) with a Rodnan skin score above 10 had 25(OH)D_3_ insufficiency compared with seven patients (17.5%) with a 10 or lower Rodnan skin score, and the difference was statistically significant. Similar to our findings, other authors found that 25(OH)D_3_ levels correlate negatively with skin involvement [28,31]. Moreover, our data show that vitamin D insufficiency is associated with a sPAP value higher than 35 mmHg. A potential connection between vitamin D deficiencies and pulmonary artery hypertension (PAH) remains undefined. Ulrich et al. reported secondary hyperparathyroidism to be prevalent in pulmonary hypertension and supposed that vitamin D deficiency may contribute to pulmonary hypertension [32]. Similarly, Demir et al. found a significant association between vitamin D deficiency and pulmonary artery hypertension [33]. One possible explanation that vitamin D might also have a potential role in the pathogenesis of PAH is that calcitriol, the active form of vitamin D, reduces the expression of the regulated hypoxia-inducible factor-1α (HIF-1α) and endothelin-1 [34]. The HIF-1α pathway has been recognized as a potential contributor to the pathogenesis of PAH [35]. Recent evidence suggests that vitamin D3 has an important effect on the maintenance of the homeostasis of B cells [36], and low levels of 25(OH)D_3_ contribute to the enhanced production of autoantibodies [37]. In our cohort, we did not observe a significant association between vitamin D_3_ insufficiency and the presence of anticentromere and anti-topoisomerase 1 autoantibodies. Our study has some limitations that must be considered in the interpretation of our findings, and the most important is the relatively small sample size. Moreover, it may be that the association found in our study is not necessarily a “cause and effect” association. However, our results are in agreement with previously reported effects of 1,25(OH)_2_D_3_ on immune cells and confirm that 25(OH)D_3_ deficiency/insufficiency could contribute to the pathophysiology of SSc [29]. Indeed, vitamin D regulates Major Histocompatibility Complex (MHC) class-II molecule expression in Antigen-Presenting Cell (APC) and the inhibition of dendritic cell maturation, and it inhibits pro-inflammatory cytokines in monocytes and macrophages and the growth of murine fibroblasts, and it has an antifibrotic action. 

## 4. Material and Methods

### 4.1. Study Population and Recruitment

We performed a cross-sectional, observational study with a control group. Forty consecutive patients with systemic sclerosis who were hospitalized to the Unit of Rheumatology in the Department of Clinical and Experimental Medicine of the University of Messina from December 2012 to March 2013 were recruited. The variants limited and diffuse of SSc were classified in according LeRoy’s criteria [38]. Forty healthy subjects matched for age and sex served as the control group. All participants were Caucasians from Southern Italy. None of the participants of the study were on supplementation with calcium and vitamin D. Patients diagnosed with other autoimmune diseases were excluded. The Local Ethics Committee for Medical Research, University Hospital of Messina has approved the study (28 May 2012); it was carried out in accordance with the Helsinki Declaration. All subjects gave their informed written consent in order to be enrolled. All SSc patients were clinically assessed at their admission in hospital. Disease duration was defined as the first non Raynaud’s symptom.

### 4.2. Clinical Parameters and Biochemical Data

Clinical data and assessment of disease activity including several variables, such acidity (pH), oxygen saturation (SO_2_), the arterial oxygen tension (PaO_2_), carbon dioxide tension (PaCO_2_), Rodnan skin score, diffusing lung capacity for carbon monoxide (DLCO), echocardiography systolic pulmonary artery pressure (sPAP), anti-nuclear antibodies (ANA), anti-topoisomerase 1 antibodies (scl70), anticentromere antibodies (ACA), heart frequency (HF), in electrocardiography PQ and QT interval were collected only in SSc patients. Disease activity were scored in a range from 0 to 10 (0 represents no disease activity, and 10 represents maximal activity), by using the criteria proposed by Valentini et al. [39]. An index ≥3 was indicative of active disease, whereas an index <3 of not-active disease [40,41,42]. Disease severity was assessed according to the Medsger’s severity score [43]. Blood samples were obtained by antecubital venipuncture between 8 and 9 a.m. after an overnight fast and a 10-min rest. The first 4–5 mL of blood were not used. Blood was collected in refrigerated vacutainers containing an anticoagulant mixture provided by Booehringer-Mannheim, immediately placed on ice, centrifuged, within a few minutes, at 2000× *g* for 20 min at 4 °C and the plasma frozen at −70 °C until assayed for 25-hydroxyvitamin D_3_ and intact parathyroid hormone (PTH). The 25-hydroxyvitamin D_3_ and PTH were measured using high-performance liquid chromatography (Bio-Rad, München, Germany). In according to 2009 ESC/ERS recommendations a value to sPAP >36 mmHg was defined “possible Pulmonary Hypertension” [44]. Vitamin D deficiency was defined as plasma levels of 25(OH)D_3_ <10 ng/mL, insufficiency between 10 to 30 ng/m and normal >30 ng/mL. The intra- and inter-assay CV were <10% for both tests.

### 4.3. Statistical Analyses

Statistical analysis were performed using Statistica 8 (Statsoft, Inc., Tulsa, OK, USA). Values are expressed as mean ± SD or percentage. Comparisons between groups were performed by Student’s *t*-test. The percentage of each variable was compared between groups by Fisher’s exact test. Chi-squared test (*x*^2^) with Yates correction was calculated to assess the individual association between independent variables and presence of insufficiency 25(OH)D_3_. Multivariate logistic regression analysis was used to adjust for confounders. Values of *p* < 0.05 were considered to statistical significant.

## 5. Conclusions

In conclusion, our data show that 50% of patients with SSc had significantly low vitamin D serum levels compared to the control group. Vitamin D insufficiency is associated with skin involvement and sPAP > 35 mmHg. However, randomized controlled trials (RCTs) on the effects of vitamin D supplementation on disease activity in patients with SSc are needed, in order to confirm the impact of vitamin D deficiency on the complex clinical features of systemic sclerosis.

## Figures and Tables

**Table 1:** Demographic characteristics of patients in the two groups; results are expressed as means ± SD and percentage.

Characteristics	SSc (*n* = 40)	Controls (*n* = 40)	*p*
Sex (Male/Female)	2/38	2/38	1.00
Age (Years)	58.47 ± 14.04	57.96 ± 12.87	0.61
Body Mass Index (kg/m^2^)	25.33 ± 4.43	24.87 ± 4.54	0.26
Smoking status (%)			
Current	8	10	0.88
Former	18	22	0.67
Never	74	70	0.82
Sunlight exposure (%)			
<5 h/week	70	65	0.67
>5 h/week	25	27.5	0.52
>10 h/week	5	7.5	0.45
Supplementation with calcium, *n*	0	0	1.00
Supplementation with vitamin D, *n*	0	0	1.00

**Table 2:** Clinical characteristics of patients with systemic sclerosis; results are expressed as means ± SD and percentage.

Characteristics	SSc (*n* = 40)
Pattern of Scleroderma, limited/diffuse (%)	12/28 (30/70)
Duration of disease (Years)	9.11 ± 6.74
Anticentromere antibodies, *n* (%)	6 (15%)
Modified Rodnan Skin Score	10.45 ± 6.74
Interstitial Lung Disease, *n* (%)	17 (44%)
Renal Crisis, *n* (%)	0 (0)
Malabsorption, *n* (%)	7 (17.5)
Musculo-skeletal involvement, *n* (%)	24 (60)
Pulmonary Hypertension, *n* (%)	9 (24%)
Creatinine (mg/dL)	0.98 ± 0.12
Total Proteins (g/dL)	7.24 ± 0.52
Albumin (g/dL)	3.88 ± 0.31
Fibrinogen (mg/dL)	287.11 ± 33.74

**Table 3:** Association between 25(OH)D_3_ insufficiency and clinical parameters in systemic sclerosis.

Variables	25(OH)D_3_ < 30 ng/mL	25(OH)D_3_ > 30 ng/mL	Test	*p*
Subset Diffuse	17 (85%)	11 (55%)	*x*^2^ Yates = 2.98	0.08
Rodnan Skin Score > 10	13 (65%)	5 (25%)	*x*^2^ Yates = 4.95	0.02 *
Digital ulcers	8 (40%)	6 (33%)	*x*^2^ Yates = 0.11	0.74
pH < 7.38	3 (15%)	0 (0%)	*x*^2^ Yates = 1.44	0.22
pO_2_ < 80 mmHg	4 (20%)	1 (5%)	*x*^2^ Yates = 0.91	0.33
pCO_2_ < 35 mmHg	4 (20%)	1 (5%)	*x*^2^ Yates = 0.91	0.33
SO_2_ < 93%	1 (5%)	1 (5%)	*x*^2^ Yates = 0.53	0.46
Systolic Pulmonary Artery Pressure > 35 mmHg	8 (40%)	1 (5%)	*x*^2^ Yates = 5.16	0.02 *
Hearth Rate < 60	1 (5%)	1 (5%)	*x*^2^ Yates = 0.53	0.46
PQ interval > 0.20 seconds	1 (5%)	0 (0%)	*x*^2^ Yates = 0.53	0.22
Long QT interval	2 (10%)	2 (10%)	*x*^2^ Yates = 0.28	0.59
Diffusing Capacity oft he Lung for Carbone Monoxide < 75%	2 (10%)	2 (10%)	*x*^2^ Yates = 0.28	0.59
Anticentromere antibodies	4 (20%)	2 (10%)	*x*^2^ Yates = 0.20	0.65
Anti SCL70 antibodies	10 (50%)	4 (20%)	*x*^2^ Yates = 2.75	0.09

* Statistically significant.
